# Population genetics of the olive‐winged bulbul (*Pycnonotus plumosus*) in a tropical urban‐fragmented landscape

**DOI:** 10.1002/ece3.1832

**Published:** 2015-12-10

**Authors:** Grace S. Y. Tang, Keren R. Sadanandan, Frank E. Rheindt

**Affiliations:** ^1^ Department of Biological SciencesNational University of Singapore14 Science Drive 4Singapore117543Singapore

**Keywords:** Birds, gene flow, genetic diversity, Old World tropics, population structure, South‐East Asia

## Abstract

With increasing urbanization, urban‐fragmented landscapes are becoming more and more prevalent worldwide. Such fragmentation may lead to small, isolated populations that face great threats from genetic factors that affect even avian species with high dispersal propensities. Yet few studies have investigated the population genetics of species living within urban‐fragmented landscapes in the Old World tropics, in spite of the high levels of deforestation and fragmentation within this region. We investigated the evolutionary history and population genetics of the olive‐winged bulbul (*Pycnonotus plumosus*) in Singapore, a highly urbanized island which retains <5% of its original forest cover in fragments. Combining our own collected and sequenced samples with those from the literature, we conducted phylogenetic and population genetic analyses. We revealed high genetic diversity, evidence for population expansion, and potential presence of pronounced gene flow across the population in Singapore. This suggests increased chances of long‐term persistence for the olive‐winged bulbul and the ecosystem services it provides within this landscape.

## Introduction

Increasing urbanization has led to a sharp increase of fragmentation worldwide. Such fragmentation may result in small, isolated populations that face great threats from demographic and environmental events (Lande 1988) as well as genetic factors (Frankham and Ralls 1998). Fragmentation may reduce genetic diversity and connectivity of populations (Young et al. 1996; Dixo et al. 2009) and may lead to an increased risk of fixation of new deleterious mutations and genetic diseases (Lande 1994), reduced survivorship and fertility (inbreeding depression) (Jimenez et al. 1994; Charlesworth and Willis 2009) and overall increased extinction risk (Saccheri et al. 1998; Brook et al. 2002). These effects have been found to affect even avian species with high dispersal propensities (Segelbacher and Storch 2002; Caizergues et al. 2003), thereby highlighting the importance of population genetic research and of maintaining genetic diversity and connectivity between fragmented populations (O'Brien and Evermann 1988).

In spite of these findings, there are relatively few population genetic studies on species living within urban‐fragmented landscapes in the Old World tropics (e.g., Campbell et al. 2006). This is alarming given that this region is also where the highest rates of deforestation and habitat degradation are occurring. While tropical rainforest loss comprised 32% of global forest cover loss from 2000 to 2012 and is estimated to increase by 2101 km^2^ per year (Hansen et al. 2013), South‐East Asia in particular has the highest annual deforestation rates across all tropical regions, and this rate has been increasing across the past two and a half decades (Sodhi et al. 2010). These figures are even more alarming given that the majority of this region falls into four biodiversity hotspots demarcated by Myers et al. (2000) and has high species richness and endemism (Sodhi et al. 2004). However, genetic work in this region has mostly focused on large‐scale relationships, such as phylogeography (Lim et al. 2011) and phylogenetic relationships (Oliveros and Moyle 2010). Many of these studies include only a few species from the Old World tropics and do not focus on any particular species (e.g., Morales and Melnick 1998; Moyle and Marks 2006; Lohman et al. 2010), much less on their population structure, patterns of gene flow or demographic changes.

In this study, we focused on Singapore (103° 50′ E, 1° 20′ N), a South‐East Asian island city situated at the southern tip of the Malay Peninsula. Singapore has lost more than 95% of its original forest cover (Tan et al. 2010). Of the forest cover that remains, <10% is primary, and the majority of it is located within the country's nature reserves in a highly fragmented state (Yee et al. 2011). Due to such deforestation, Singapore has suffered catastrophic extinctions across numerous taxa and lost many species, with several more designated as “living dead” (Brook et al. 2003). Even with a conservative estimate, 41 bird species have gone extinct on the island within just two centuries, 24.4% of which were frugivorous (Wang and Hails 2007). While population genetics studies of the island's remaining wildlife would directly impact conservation of Singapore's biodiversity, more importantly, such studies would have larger implications for the long‐term persistence of the same species or members of the same ecological guild as well as their ecosystem services in other urban‐fragmented landscapes.

**Figure 1:**
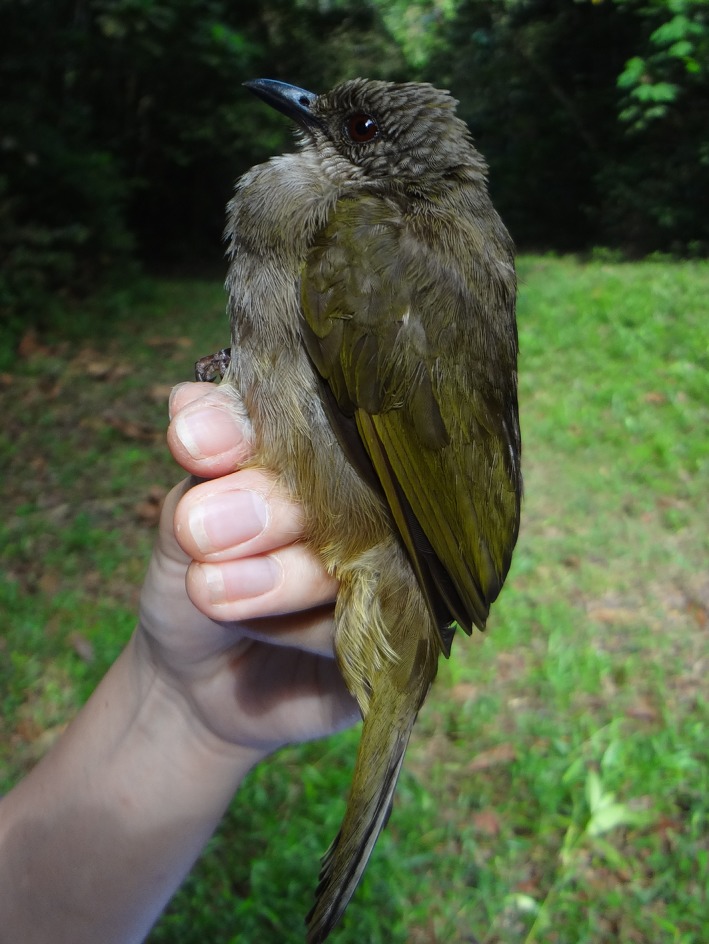
Singaporean specimen of olive‐winged bulbul (*Pycnonotus plumosus*).

In Singapore, the olive‐winged bulbul (*Pycnonotus plumosus*, Fig. 1) is a nonmigratory native species which feeds on small fruits and berries, beetles, and small insects (Whittaker and Jones 1994). As an obligate frugivore, it has been documented consuming fruit, seed, and even seed arils from several plant species found in Singapore (Briffett 1993; Kelvin and Chong 2003; Wells 2007), suggesting that it is an important resident seed disperser. Additionally, *P. plumosus* is a species that is not forest‐dependent and is widespread and common throughout Singapore; although no official local population estimate has been made, annual bird censuses have consistently recorded its presence across a 10‐year period at 17 of 33 sites (51.52%) surveyed, although reportedly at a declining overall population trend (Lim and Lim 2009). As a forest‐edge species found mainly in secondary forest and scrub, parkland, but also in coastal and mangrove vegetation (Wells 2007; Yong et al. 2013), *P. plumosus* could therefore facilitate seed exchange between forest fragments of differing quality and levels of isolation. The species could therefore be a very important mobile link and habitat restoration agent for the majority of Singapore's remaining forests that exist as isolated fragments across a heterogeneous landscape, in comparison with other species which are habitat specialists (Wunderle 1997).

We investigated the population genetics and evolutionary history of *P. plumosus* in Singapore, with reference to other populations in South‐East Asia. We analyzed novel, single‐gene sequences for 49 *P. plumosus* individuals from Singapore together with GenBank sequences from the region for a total of 80 samples. A smaller subset of Singaporean samples were then further analyzed at three mitochondrial loci. Our aims were to investigate *P. plumosus* genetic diversity, population genetic structure, and patterns of gene flow within Singapore and to elucidate and place the evolutionary history of Singaporean *P. plumosus* in a regional perspective. Specifically, we wished to determine whether the levels of genetic diversity and gene flow across the population in Singapore had been adversely affected by the high level of habitat fragmentation.

## Methods

### Sample collection and sequencing

**Figure 2:**
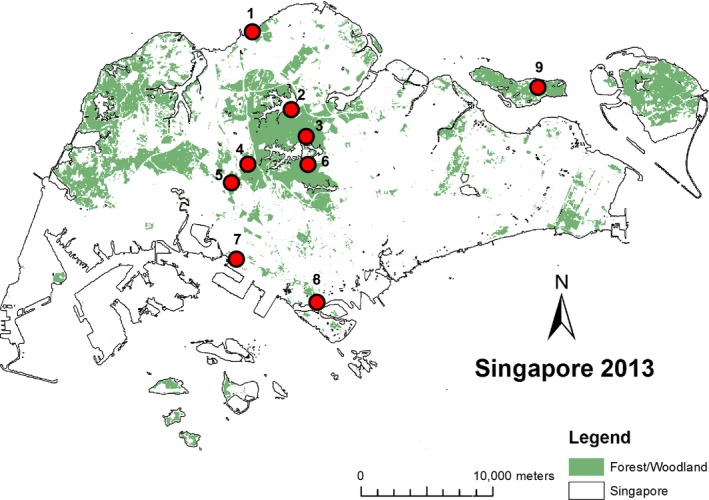
Map of Singapore showing sampling localities (red circles) within the urban‐fragmented landscape (Tan and Rheindt 2015). Numbers next to sampling localities correspond to locality numbers in the sample list (see Table S1). Mist netting was also conducted in Pasir Ris Park (eastern Singapore), but no target samples were obtained in that location, and it is therefore not shown here.

Blood samples were obtained from birds captured using mist nets (6, 12 or 18 m × 2.5 m, mesh size 30 mm) between end of April 2013 and mid‐January 2014 in various locations across Singapore (Fig. 2). *Pycnonotus plumosus* is resident and nonmigratory, and hence, data collected during this period would not be affected by seasonal movement patterns. For detailed sample locality information, see Table S1.

Feather or skin samples were collected opportunistically. Details of sample collection are provided in Appendix S1. A total of 39 target samples were obtained across Singapore over a total of 2128.32 mist‐netting hours. Additionally, 10 tissue samples were also obtained from the Raffles Museum of Biodiversity Research (Singapore) (meanwhile renamed the Lee Kong Chian Natural History Museum) and the Burke Museum of Natural History and Culture (Seattle, Washington).

Three mitochondrial coding genes were examined – cytochrome c oxidase subunit I (COI), NADH dehydrogenase subunit 2 (ND2), and cytochrome‐*b* (cyt‐*b*). DNA extraction from field‐obtained samples was initially carried out using target blood samples. However, visualization of the PCR products in 0.8% agarose gel consistently gave smears or multiple bands. Following this, all DNA extractions were carried out using feather samples instead of blood, using the GeneAll^®^ Exgene^™^ Clinic SV mini kit, following the manufacturer's protocol for hair. DNA extraction from the 10 museum‐sourced tissue samples was conducted using either the GeneAll^®^ Exgene^™^ Clinic SV mini kit following the manufacturer's protocol for animal tissue, or via phenol/chloroform extraction (Sambrook and Russell 2006), depending on tissue quality.

Polymerase chain reaction (PCR) was then conducted on C1000^™^ Thermal Cyclers (Bio‐Rad Laboratories, Inc., Hercules, CA) for COI, ND2, and cyt‐*b*. Detailed laboratory procedures can be found in Appendix S1. Post‐PCR products were purified using ExoSAP‐IT^®^ (Affymetrix, Inc., Santa Clara, CA), and cycle‐sequenced using the BigDye^®^ Terminator v3.1 Cycle Sequencing Kit (Applied Biosystems Inc., Foster City, CA) and the same primers used for PCR. Cycle‐sequenced products were then purified using ALINE PureSEQ Solution (Aline Biosciences, Woburn, MA) following the manufacturer's protocol and subsequently sequenced in an ABI 3130xl Genetics Analyzer (Applied Biosystems). Sequences were then blasted in GenBank to confirm species identity, and reconciled into gene sequence contigs using CODONCODE ALIGNER v4.2.3 (CodonCode, Dedham, MA). Contigs from the three genes were concatenated and were subsequently aligned using the ClustalW option in Mega 5.2 (Tamura et al. 2011).

### Genetic analysis

Upon inclusion of preexisting GenBank sequences, two subsets of data were defined to maximize number of individuals and number of loci examined, respectively. The first dataset – based on ND2 alone – contained the largest number of sequences upon inclusion of 31 GenBank ND2 *P. plumosus* sequences from South‐East Asia, generating a total of 80 target sequences from throughout the region (see Table S1 for GenBank sequence information). The second dataset comprised freshly obtained sequences spanning all three mitochondrial genes (COI, ND2, and cyt‐*b*) (henceforth three‐gene dataset). Due to issues with cyt‐*b* amplification, this dataset had a reduced representation comprising 29 individuals all of Singaporean origin.

For all subsequent analyses except the phylogenetic analyses (which are able to deal with missing data), all sites with gaps or ambiguity codes were removed across all samples. The final ND2 dataset comprised 77 individuals spanning 825 base pairs, while the concatenated three‐gene dataset, giving 29 individuals spanning 1812 base pairs. Details for each analysis are provided in Appendix S1.

To investigate haplotypes in the dataset, GenAlEx 6.501 (Peakall and Smouse 2006, 2012) was used to determine the number of haplotypes in each dataset and NETWORK v4.6.1.2 (Fluxus Technology Ltd., Clare, Suffolk, England) was used to construct the haplotype network using the median‐joining algorithm.

To reconstruct genetic relationships, neighbor‐joining (NJ), maximum parsimony (MP), and maximum likelihood (ML) analyses were independently carried out for each data subset, using Mega 5.2 (Tamura et al. 2011). For each analysis, the short‐tailed babbler (*Pellorneum malaccense*) was used as the out‐group taxon, following recent phylogenetic studies showing babblers to be close relatives of bulbuls (*Pycnonotidae*) (Barker et al. 2004; Alström et al. 2013). jModelTest (v 2.1.4) (Guindon and Gascuel 2003; Darriba et al. 2012) was used to determine the best model for the ML analysis of each dataset.

To compute various population genetic parameters, analyses of molecular variance (AMOVA) were carried out on both datasets in ARLEQUIN v3.5.12 (Excoffier and Lischer 2010). The ND2 dataset was used in AMOVA to investigate variation among geographical regions, while the three‐gene dataset was used to investigate variation among Singaporean sampling localities. Mismatch distribution analysis was also conducted in ARLEQUIN v3.5.12 under the model of demographic expansion. This analysis was only performed on the ND2 dataset and for Singapore and Johor (as one group), Sarawak, and Sabah. Analysis for Palawan was not carried out due to small sample size (three individuals).

Using the ND2 dataset, default settings in DnaSP v5.10.01 (Librado and Rozas 2009) were then used to compute values of nucleotide diversity (*π*) for each of the five geographical regions (Singapore, Johor, Sarawak, Sabah, Palawan), and mean p‐divergence values between the five geographical regions. MEGA 5.2 (Tamura et al. 2011) was then used to compute raw pairwise p‐divergences within each of three geographical regions (Singapore, Sarawak, and Sabah). Analyses of pairwise differences were not run for Johor or Palawan due to the small sample size (one individual and three individuals, respectively). Graphs of pairwise differences in base pairs were then constructed using the output from MEGA 5.2.

In order to contrast Singaporean haplotype diversity with comparable regional populations, sub‐sampling and rarefaction techniques were used. We compared the Singaporean olive‐winged bulbul population with the population of short‐tailed babblers in Singapore (Sadanandan and Rheindt 2015), as well as with the Philippine bulbul population in Mount Apo (Campbell 2013), an area of similar size to Singapore. The populations were sub‐sampled and rarefied as per Gotelli and Colwell (2001) using 20 bootstrap replicates in each case. DnaSP v5.10.01 was used to calculate and contrast levels of genetic diversity in Olive‐winged bulbuls, Philippine bulbuls, and Short‐tailed babblers.

## Results

The final ND2 dataset used primarily for regional haplotype analysis comprised 77 individuals spanning 825 base pairs of sequence. Of these 825 sites, 133 were polymorphic (i.e., single nucleotide polymorphisms, or SNPs), giving rise to a total of 28 haplotypes across the region. Of these, 15 belonged to the Singaporean population, five belonged to the Sabah population, five belonged to the Sarawak population, and one belonged to the Palawan population. The single remaining haplotype was shared between Sabah and Sarawak. The final concatenated three‐gene dataset used for the Singaporean haplotype analysis comprised 29 samples. This dataset spanned 1812 base pairs, of which 33 were SNPs. Analysis of this dataset revealed 16 haplotypes within the Singapore population, seven of which were shared across more than one locality.

### Singapore‐specific analysis of genetic diversity

**Figure 3:**
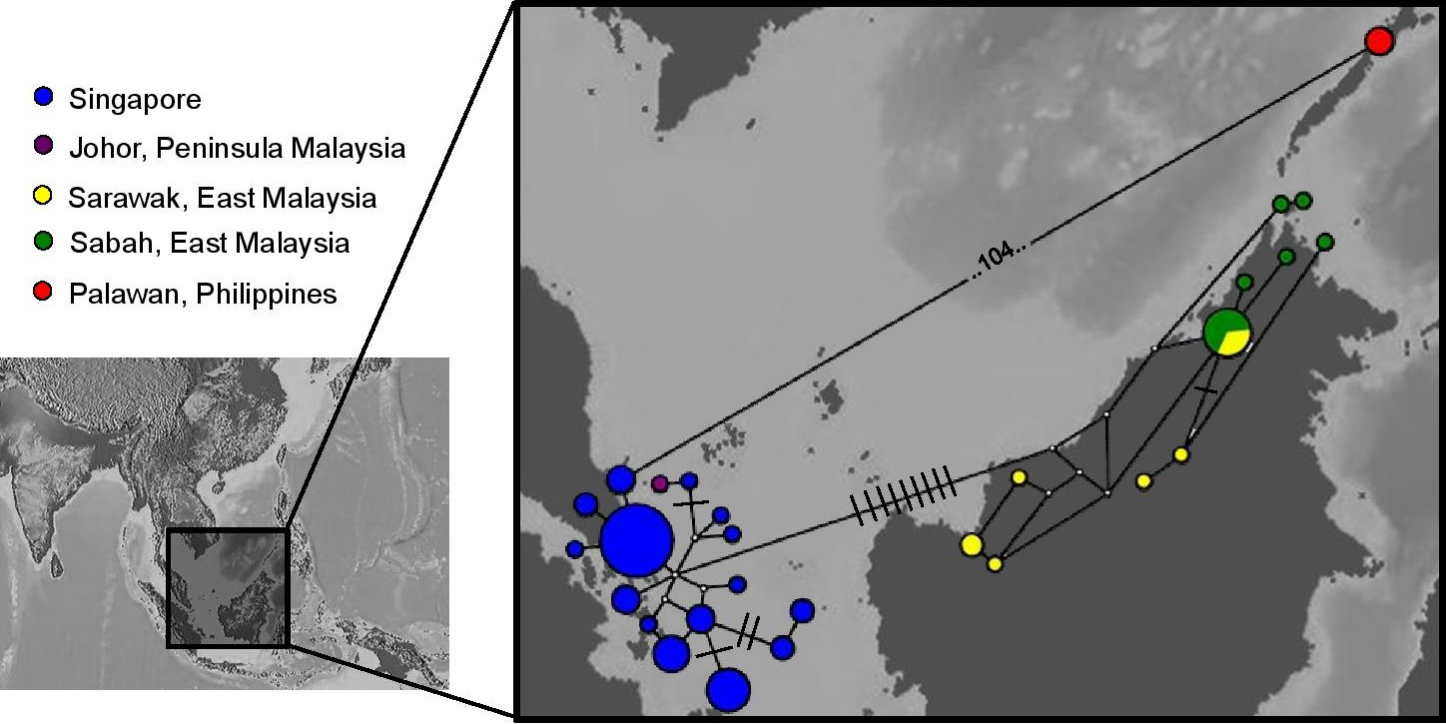
Haplotype network constructed using ND2 sequences across the region. The size of the circle is proportional to the number of individuals sharing that haplotype. Lines between adjacent circles represent one base pair of a difference from one haplotype to the other, unless indicated otherwise by horizontal bars or numerical values. Unfilled circles represent median vectors (unsampled haplotypes). Three deeply diverged clusters are obtained corresponding to the geographical regions of Singapore and Johor, Borneo, and Palawan.

In the haplotype network (Fig. 3), the Singaporean olive‐winged bulbuls were found to form a cluster comprising 15 distinct haplotypes of varying frequencies. The difference between the two most different haplotypes of this cluster (excluding the sample from Johor), that is, the maximum p‐divergence within Singapore (Table 1), was nine base pairs.

**Table 1:** Matrix of nucleotide diversity, *π* (above diagonal), and mean p‐divergence (below diagonal), among the five geographical regions (Singapore, Johor, Sarawak, Sabah, and Palawan). Values in the diagonal (gray) are intraregion p‐divergence ranges

	Singapore	Johor	Sarawak	Sabah	Palawan
Singapore	0.0000–0.0121	0.0041	0.0078	0.0083	0.0169
Johor	0.0073	N.A.^a^	0.0068	0.0055	0.0667
Sarawak	0.0194	0.0205	0.0000–0.0085	0.0031	0.0559
Sabah	0.0192	0.0214	0.0034	0.0000–0.0085	0.0491
Palawan	0.1290	0.1333	0.1321	0.1315	0.0000

a p‐divergence values could not be calculated for Johor due to small sample size (one sample).

**Figure 4:**
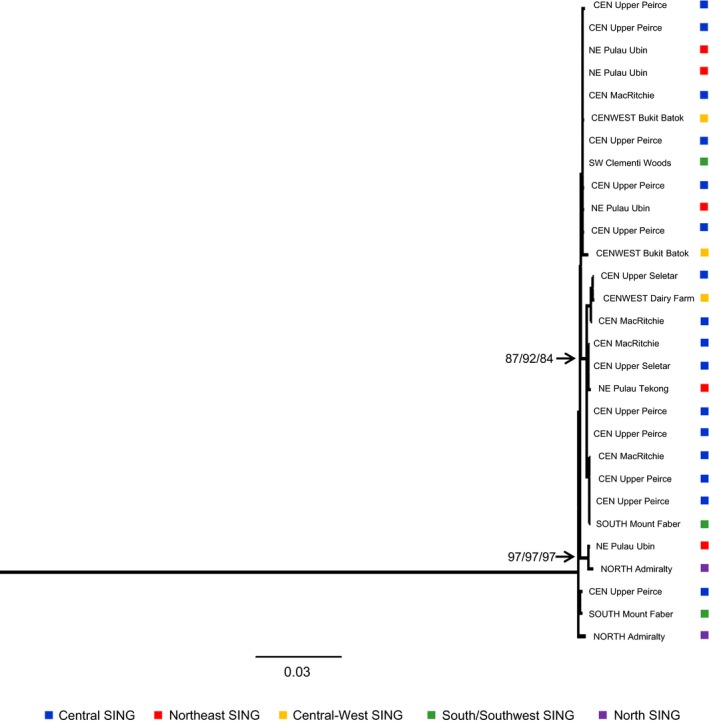
Maximum likelihood (ML) phylogram constructed from the three‐gene dataset of the olive‐winged bulbul (out‐group removed). ML produced the phylogram with the most number of nodes with high bootstrap support (>90) and is thus depicted. Bootstrap values are shown for nodes with values >90. The three numbers shown for each node represent values derived from the three phylogenetic analysis methods – ML, maximum parsimony, and neighbor‐joining, respectively – for that node. SING stands for Singapore. Capitalized words preceding locality name indicate general location of locality in Singapore: CEN for stands central, NE for northeast, CENWEST for central‐west, and SW for southwest.

Singapore‐specific phylogenetic analysis produced topologies congruent across all three analytical methods (NJ, MP, and ML) (Fig. 4). In this topology, samples from particular localities or even general localities (such as central or south Singapore) did not form separate, strongly supported clades. Similarly, the two strongly supported nodes (bootstrap values >90) consisted of samples from different localities. The haplotype map based on Singaporean ND2 data (Fig. S1) similarly revealed that haplotypes were not segregated by locality.

This result was further supported by the population values of nucleotide diversity (*π*) and p‐divergence among the five general collecting localities of Singapore (Table S2), and the results from AMOVA using Singaporean samples (Table S3). The *π* and p‐divergence values were generally similar across localities, although p‐divergence values between individuals in the northern part of Singapore and those of other Singaporean localities (0.00436–0.00543) were much higher than p‐divergence values between other localities (0.00268–0.00325) (Table S2). Similarly, AMOVA attributed almost all of the variation to within‐locality variation (91.52%), and very little to among‐locality variation (8.48%) (Table S3). The nonsignificant *P*‐value among localities (0.094) and the low *F*_st_ estimate (0.08) further indicate that differences among localities were insignificant.

### Population genetic comparison

**Figure 5:**
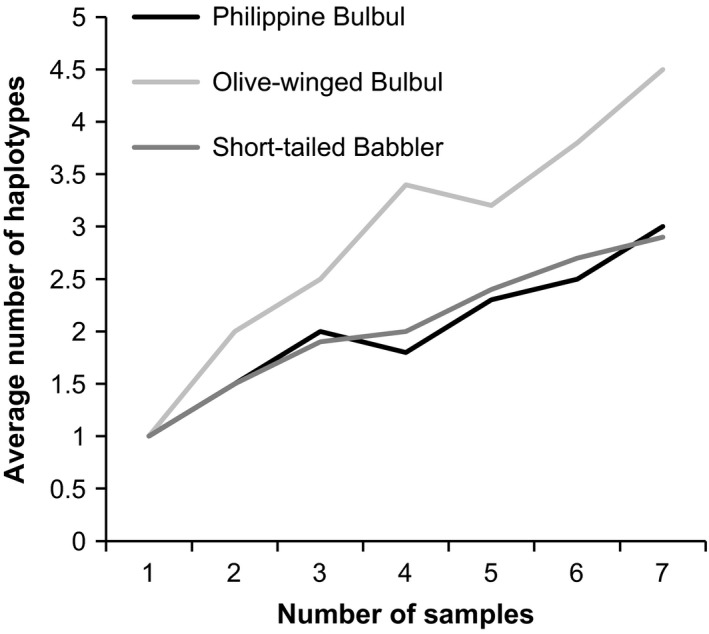
Rarefaction curve to compare haplotype diversity between olive‐winged bulbuls in Singapore, Philippine bulbuls in Mount Apo, and short‐tailed babblers in Singapore based on ND2 sequences.

Comparison of genetic diversity between olive‐winged bulbuls, Philippine bulbuls, and short‐tailed babblers, corrected for sample size and using individual‐based rarefaction curves, revealed that olive‐winged bulbuls showed a much greater rate of haplotype accumulation per sample (Fig. 5). Levels of nucleotide diversity and haplotype diversity were over triple and almost double, respectively, than that seen in the Philippine bulbul (Table 2). Furthermore, Chao1 analysis of Singaporean olive‐winged bulbul haplotypes based on the number of singleton and doubleton haplotypes produced a haplotype estimate of 36.1. The observed number of haplotypes in the population was 16 – the difference in these figures indicates that there is probably a high proportion of unsampled diversity in the Singaporean population.

**Table 2:** Comparison of genetic diversity markers across selected taxa

	Nucleotide diversity, *π*	Number of polymorphic sites	Average number of nucleotide differences, k	Haplotype diversity, Hd
Olive‐winged Bulbuls, Singapore (*n* = 55)	0.00325	12	1.880	0.802
Short‐tailed Babblers, Singapore (*n* = 33)	0.00084	2	0.530	0.483
Philippine Bulbuls, Mount Apo (*n* = 7)	0.00135	3	0.857	0.524

### Region‐wide analysis of evolutionary history

Haplotype networks constructed using the region‐wide ND2 dataset found three main clusters, corresponding to the same three regions of Singapore and Johor versus Borneo and versus Palawan (Fig. 3). The one individual from Johor was placed in the same cluster as the Singaporean individuals. A large number of distinct haplotypes of varying frequencies was obtained for each cluster, such as the Bornean cluster which possessed 11 haplotypes. No one haplotype was found to dominate the populations in these regions, although some were more frequent than others.

**Figure 6:**
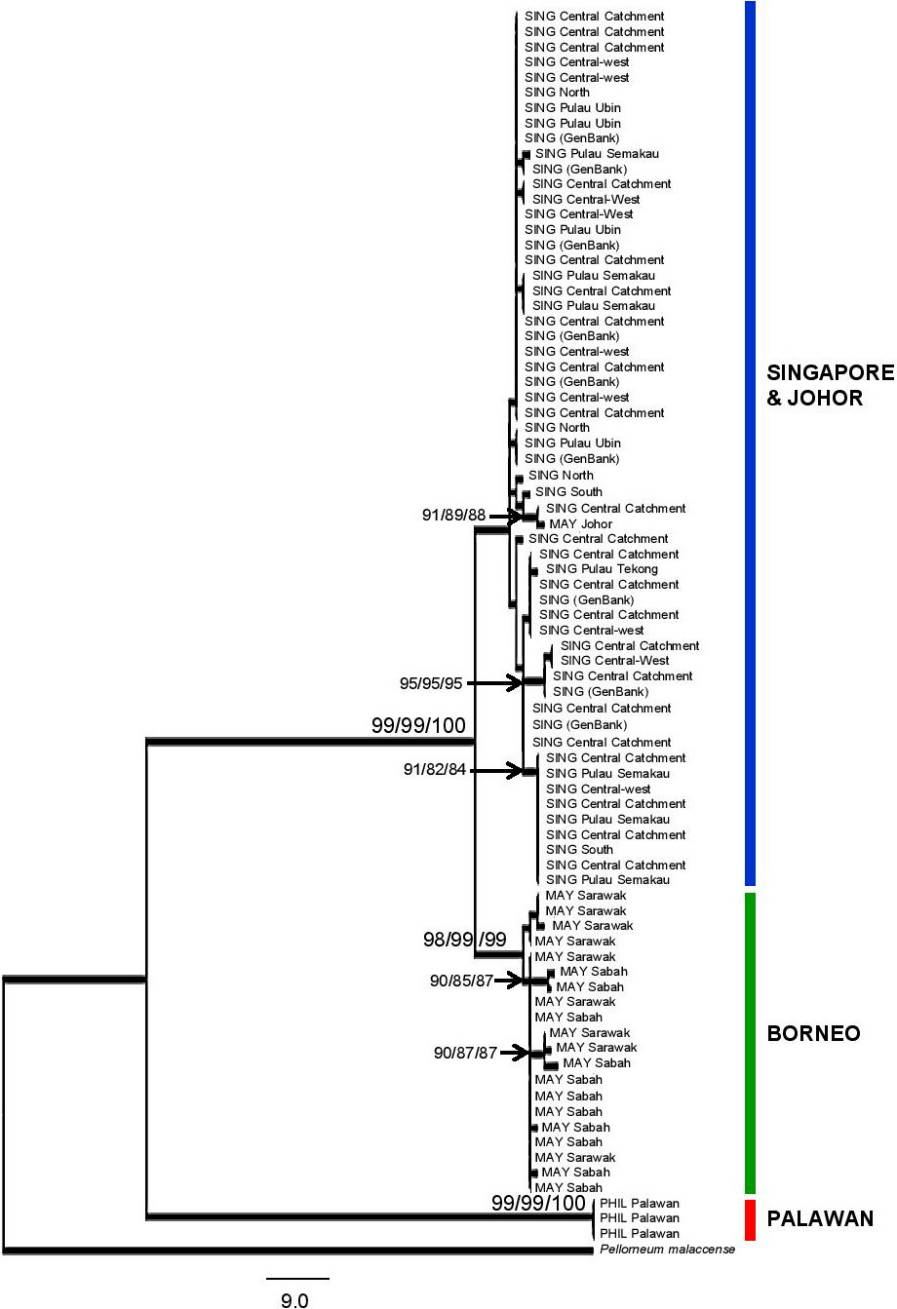
Maximum parsimony (MP) phylogram constructed from ND2 sequences of *P. plumosus* and the out‐group (*Pellorneum malaccense*). MP produced the phylogram with the most number of nodes with high bootstrap support (>90) and is depicted here. Bootstrap values are shown for nodes with values >90. The three values at a node represent those derived from the three phylogenetic analysis methods – MP, maximum likelihood, and neighbor‐joining, respectively – for that node.

Additionally, all three phylogenetic analytical methods (NJ, MP, and ML) produced virtually the same topologies (Fig. 6), with deep divergences among the three geographical regions (Singapore + Johor, Borneo, and Palawan). Populations of each geographical region formed distinct clades, and each of these clades was strongly supported by high (>90) bootstrap resampling values in all three analytical methods. Additional nodes also received strong bootstrap support by one or all of the three analytical methods. The Palawan samples were shown to be exceptionally genetically diverged from other regional populations, with a ~13% mean p‐divergence between itself and other sampled areas (Singapore, Johor, and Borneo; Table 1). In contrast, the highest p‐divergence seen between the any of the other sampled populations was 2.1% (between Sabah and Johor).

For AMOVA, small sample size would not allow the analysis to be performed for Johor as a separate geographical region. The one individual from Johor was therefore grouped with samples from Singapore, a decision also evolutionarily accurate as shown by results of the previous phylogenetic and haplotype network analyses (Figs. 3 and 5). AMOVA was conducted on the four geographical regions and revealed large regional differences. Differences among regions or groups accounted for a larger percentage of variation (88–90%) compared to differences within regions or groups (9–10%) while F_*st*_ estimates were also high (0.90) among the three regions (Table 3).

**Table 3:** Analysis of molecular variance (AMOVA) and fixation indices of three groups of olive‐winged bulbul populations: Singapore and Johor, Borneo (comprising Sarawak and Sabah), and Palawan

Hierarchical structure	Source of variation	Sum of squares (df)	Variance component	*P*‐value	Percentage of variation	Fixation index
1	Among groups	487.448 (2)	14.02	0.066	88.24	*F*_CT_ = 0.88
2	Among populations within groups	7.665 (2)	0.40	0.001	2.51	*F*_SC_ = 0.21
3	Within populations	105.913 (72)	1.47	<0.00001	9.26	*F*_ST_ = 0.91

**Figure 7:**
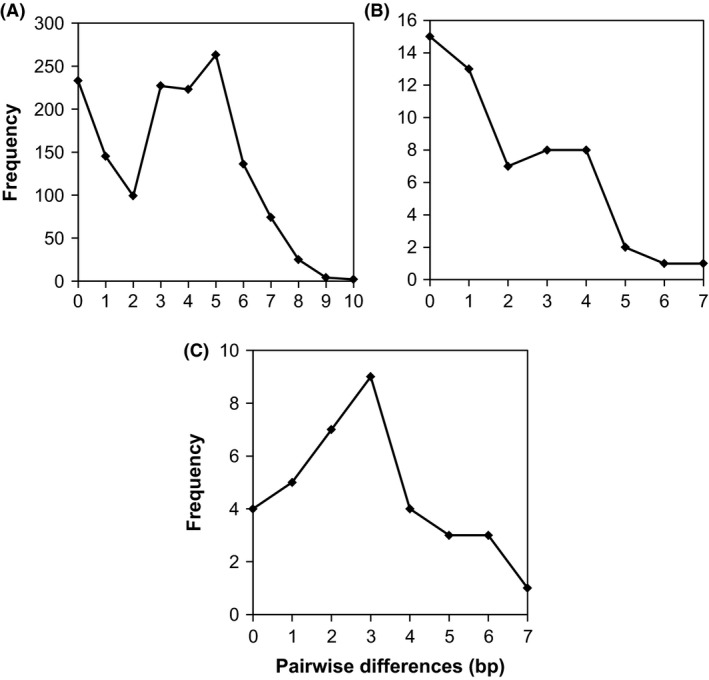
Clockwise from top left (A–C): Frequency distribution of pairwise sequence differences among olive‐winged bulbul individuals from Singapore and Johor, from Sarawak, and from Sabah, respectively.

Mismatch distribution analysis resulted in nonsignificant *P*‐values for both tests of goodness of fit (Harpending's raggedness index test and SSD) (Table 4), indicating no significant difference between the observed data and data expected under the model of sudden demographic expansion. Additionally, plots of pairwise differences for these three regions were roughly unimodal (Fig. 7). The maximum pairwise difference in base pairs was nine for the Singaporean population alone, and 10 when the sample from Johor was included in the analysis. For the populations in Sabah and Sarawak, the maximum pairwise difference in base pairs was seven in both cases.

**Table 4:** Mismatch distribution analysis results for olive‐winged bulbul populations, grouped by geographical region

Group	τ (Low bound; Upper bound)	*θ*_*0*_ (Low bound; Upper bound)	*θ*_*1*_ (Low bound; Upper bound)	Raggedness Index (*P‐*value)	SSD (*P‐*value)
Singapore and Johor	4.902 (1.174; 8.525)	0.000 (0.000; 1.876)	8.043 (4.415; 99999)	0.02475 (*P *>* *0.05)	0.01103 (*P *>* *0.05)
Sarawak	3.129 (0.830; 5.875)	0.109 (0.000; 1.512)	12.891 (3.573; 99999)	0.03086 (*P *>* *0.05)	0.00614 (*P *>* *0.05)
Sabah	3.711 (0.000; 7.461)	0.002 (0.000; 0.872)	2.741 (0.285; 99999)	0.02612 (*P *>* *0.05)	0.00602 (*P *>* *0.05)

Note: Parameters were estimated under a model of sudden demographic expansion for each geographical location. Values in parentheses refer to 95% confidence intervals, where *α *= 0.05, calculated using 1000 bootstrap replicates.

## Discussion

Analysis of the Singaporean olive‐winged bulbul population revealed a high level of genetic diversity (Table 2; Fig. 5), with no evidence of past population bottlenecks (Table 4). The absence of clear population partitioning by localities also suggests that significant amounts of gene flow potentially occur across the entire Singaporean population. On the other hand, our region‐wide analysis of evolutionary history corroborated deep divergences among populations in the three geographical regions of Singapore (including Johor), Borneo, and Palawan, with the latter emerging as exceptionally genetically distinct (Oliveros and Moyle 2010; Lim et al. 2011). Our findings have important implications for the long‐term population persistence of local and regional olive‐winged bulbul populations as well as the ecosystem services they provide.

### Outlook for Singaporean population

Our findings suggest a positive outlook for the Singaporean olive‐winged bulbul population. The population's intraspecific genetic divergence, at approximately 1.1% (or nine of 825 base pairs), is vast for an island only slightly more than 710 km^2^ in size (Tan et al. 2010). This value is in fact three times larger than conventional intraspecific divergences of 0.23–0.27% found for hundreds of other bird species, including individuals across large geographical areas many times the size of Singapore (e.g., Hebert et al. 2004; Kerr et al. 2009). Additionally, comparisons of genetic diversity with the two comparison populations – short‐tailed babbler population in Singapore and the Philippine bulbul population in Mount Apo, an area of similar size to Singapore – revealed that the Singaporean olive‐winged bulbul population has the highest genetic diversity of the three. This extremely high genetic diversity implies that the population is better equipped to deal with environmental and genetic events, allowing for better chances of long‐term persistence than a genetically depauperate species. Further, the results of the Chao1 analysis indicate that there are many unsampled haplotypes, and that an even higher level of genetic diversity could be uncovered with greater sampling effort and higher resolution data spanning more loci and linkage groups.

Additionally, the diverse haplotype network cluster, the nonsignificant *P‐*values from mismatch distribution analysis and the roughly unimodal plots of pairwise differences suggest that the population in Singapore has undergone a recent population expansion. This finding is in agreement with the widespread deforestation that has occurred or is occurring in this region (Sodhi et al. 2004, 2010) and the resultant spread of forest‐edge habitat utilized by olive‐winged bulbuls. It also suggests that the population in fact expanded from one with high genetic diversity. Genetic variation within this population therefore appears to be long‐standing. Our study is unique in that it shows a native species expanding in response to fragmentation; most cases of population expansion involve introduced species, such as house sparrows in Kenya (Schrey et al. 2013).

This suggestion is further corroborated by the high level of genetic diversity found within the Singaporean population. Going by the avian mitochondrial molecular clock rate of 2.1% (Weir and Schluter 2008), the level of genetic divergence (1.1%) within the Singaporean *P. plumosus* population would have taken at least half a million years to accumulate. This indicates a long‐term occurrence of the species on the island long before human settlements, when the species' preferred habitat would have been riverbanks, coastal scrub, mangrove forest, as well as edges created naturally through the action of megafauna, natural treefalls, or the spread of savannah‐like vegetation into the region during this period (Bird et al. 2005).

As a final contribution to the species' positive outlook in Singapore, the absence of population structuring by localities suggests that gene flow has been occurring across localities in Singapore and may be occurring across the entire island (including offshore islands). As the species is nonmigratory, this pattern of gene flow might be reflective of a baseline, year‐round pattern within the local population, unaffected by migratory individuals or seasonal movements. This finding also stands in contrast to other Singaporean species such as the short‐tailed babbler (*Pellorneum malaccense*) (Sadanandan and Rheindt 2015), as well as to other avian populations on islands, peninsulas and even large geographical areas (e.g., Blakiston's fish owl [*Bubo blakistoni*] (Omote et al. 2015), Spanish imperial eagle [*Aquila adalberti*] (Martínez‐Cruz et al. 2007), white‐breasted wood‐wren [*Henicorhina leucosticte*] (Brown et al. 2004), Siberian jay [*Perisoreus infaustus*] (Uimaniemi et al. 2000)) which all display impoverished population genetic diversity and a lack of gene flow across their respective urban‐fragmented landscapes. Higher resolution data with more samples would reveal whether the population is not only genetically diverse but also potentially near‐panmictic, as current data suggest.

Ecologically, these findings offer hope for long‐term persistence of *P. plumosus* and its ecological services in Singapore. The population's demographic expansion indicates that olive‐winged bulbuls now have an even greater contribution to ecosystem services in these regions. As important mobile links within an increasingly fragmented landscape, their potential to connect and restore degraded forest habitat has, and will, become even more significant in mitigating the effects of the same deforestation that purportedly benefits them demographically. However, given that these populations have high genetic diversity and gene flow potentially occurring across the island, such services appear to be sustainable over a longer time‐course, offering hope that preexisting or continued damage to fragmented landscapes may be mediated to some extent in the long run.

### Evolutionary history of regional populations

Deep divergences among regional *P. plumosus* populations suggest that gene flow among these populations is most likely no longer occurring. Following the avian mitochondrial molecular clock rate of 2.1% (Weir and Schluter 2008), the 13% divergence between Singapore and Palawan and between Borneo and Palawan translates to a divergence of at least 6.1 million years between Palawan and other populations, beginning in the Miocene. In contrast, Singaporean and Bornean populations have experienced more recent mitochondrial exchange, yet the divergence of nearly 2% is still considerable, and dates to approximately one million years ago during the Pleistocene. This implies that gene flow between the Bornean and Singaporean populations may have already ceased to occur even when the populations were connected, such as during most recent glacial maxima in the Pleistocene.

As a forest‐edge species which can tolerate varying habitat conditions, it is unlikely that unsuitable vegetation barriers impeded gene flow of *P. plumosus* across the land bridge connection (Bird et al. 2005; Cannon et al. 2009; Lim et al. 2011). Reasons for divergence of the Singaporean, peninsular Malaysian, and Bornean populations could then be that the populations in these regions had diverged too far to continue interbreeding even when land connections were present and gene flow possible. However, as these findings are based on a single maternally inherited linkage group, such conclusions would definitely benefit from further work with multigene datasets and additional samples from other Sundaic regions.

Mismatch distribution analysis results (Table 4) and plots of pairwise differences (Fig. 7) in support of a recent population expansion were similarly obtained for the Bornean populations. These findings constitute an optimistic projection for the species' long‐term persistence in these increasingly deforested regions (Sodhi et al. 2004, 2010), with larger, genetically diverse populations possessing great capacity to buffer against stochastic events which may become more common with climate change and increasing habitat fragmentation (Palmer and Räisänen 2002). Given that this study was conducted using a single maternally inherited linkage group, future work involving additional loci and other taxa would allow for a more detailed picture of the population genetics and long‐term persistence of key species in urban‐fragmented landscapes, with implications for wildlife conservation.

## Conflict of Interest

None declared.

## Supporting information


**Appendix S1**. Details of sample collection and sequencing, as well as genetic analysis procedures.

**Figure S1.** Haplotype map of Singapore based on ND2 data, showing haplotypes by locality.
**Table S1.** Sample number and collection locality of study samples.
**Table S2.** Matrix of nucleotide diversity, showing π (above diagonal) and p‐divergence (below diagonal) among the five general collecting localities of Singapore (Central Catchment, Central‐west Singapore, South and Southwest Singapore, Northern Singapore, and Northeast offshore islands).
**Table S3.** Analysis of molecular variance (AMOVA) of Singaporean olive‐winged bulbul populations, grouped into five general collecting localities (Central Catchment, Central‐west Singapore, South and Southwest Singapore, Northern Singapore, and Northeast offshore islands).
