# Supplementary material for: Population genetics of the olive‐winged bulbul (Pycnonotus plumosus) in a tropical urban‐fragmented landscape
Source: Ecol Evol. 2015 Dec 10;6(1):78–90. doi: 10.1002/ece3.1832 (PMC4716506; doi:10.1002/ece3.1832)
Supplement: Supplementary file 1 — Appendix S1. Details of sample collection and sequencing, as well as genetic analysis procedures. [file ECE3-6-078-s001.docx]

**Appendix S1:** **Details of sample collection and sequencing, as well as genetic analysis procedures.**

*Sample collection and sequencing*

Birds captured using mist nets were extracted from the net and banded with a numbered ring. Prior to taking samples, the skin surrounding the basilic vein was sterilised using 100% ethanol. Blood samples were then obtained from the birds via venipuncture of the basilic vein using a sterile BD PrecisionGlide™ needle (0.3 mm × 13 mm) and 50 μl Drummon™ Short-Length Microcap™ heparin-free capillary tubes. Blood samples were stored in 100% ethanol to a dilution factor of 10 to 20.

DNA extraction from field-obtained samples was initially carried out using target blood samples, and was performed using the GeneAll® Exgene™ Clinic SV mini kit, following the manufacturer’s protocol for blood and body fluid using microcentrifuge. Polymerase chain reaction (PCR) was then conducted on C1000™ Thermal Cyclers (Bio-Rad Laboratories, Inc., California, USA) for COI, using the thermal profile and primers—BirdF1 and BirdR2—from Hebert et al. (2004). PCR reactions of ND2 and cyt-b followed the thermal profile from Dong et al. (2010a); ND2 fragments were amplified using primers L5219Met and H6313Trp (Sorenson et al. 1999), while cyt-b fragments were amplified using primers L14833 (Dong et al. 2010b) and H14857 (Cibois et al. 1999). However, visualisation of the PCR products in 0.8% agarose gel consistently gave smears or multiple bands. Following this, all DNA extractions were carried out using feather samples instead of blood. DNA extraction from feathers was performed using the GeneAll® Exgene™ Clinic SV mini kit, following the manufacturer’s protocol for hair. DNA extracted from tissue and from feathers was then amplified using the same PCR protocols.

*Genetic analysis*

Phylogenetic analyses were conducted without any removal of gaps or ambiguity codes in the sequences. Neighbour-joining (NJ) analyses in this study were conducted with 10,000 bootstrap replicates using raw p-distances and a pairwise-deletion treatment of missing data. Maximum parsimony (MP) analyses were conducted with 1000 bootstrap replicates on all sites using Tree-Bisection-Reconnection with default settings. For maximum likelihood (ML) analyses, jModelTest (v 2.1.4) (Guindon and Gascuel 2003; Darriba et al. 2012) gave the Hasegawa-Kishino-Yano model with invariant sites (HKY+I) (Hasegawa et al. 1985) as the best model in both cases. Maximum likelihood (ML) analyses for each dataset were run using the HKY+I model, using all sites for 1000 bootstrap replicates and using default settings for tree inference options and system resource usage.

For all subsequent analyses, all sites with gaps or ambiguity codes were removed across all samples. Due to long leading or trailing gaps, three samples (M0938, CSW8481, and KLE621) were removed entirely from the ND2 dataset, leaving 77 individuals spanning 825 base pairs. No individuals were removed from the concatenated three-gene dataset.

To construct the haplotype network, DnaSP v5.10.01 (Librado and Rozas 2009) was first used to convert the datasets into the Roehl data format (.rdf) for input into NETWORK v4.6.1.2 (Fluxus Technology Ltd.). The haplotype network was then constructed using the median-joining algorithm with no modification of the network torso.

To compute various population genetic parameters, DnaSP v5.10.01 was used to reformat the datasets for processing in ARLEQUIN v3.5.12 (Excoffier and Lischer 2010). Analyses of molecular variance (AMOVA) were then carried out on both datasets in ARLEQUIN v3.5.12 using the settings of standard AMOVA computations and 1000 permutations. Mismatch distribution analysis was conducted in ARLEQUIN v3.5.12 under the model of demographic expansion using pairwise differences and 1000 bootstrap replicates.

The computation of raw pairwise p-divergences within each of three geographical regions (Singapore, Sarawak, and Sabah) using MEGA 5.2 (Tamura et al. 2011) was done with additional settings of 1000 bootstrap replicates and pairwise deletion of gaps or missing data.

**References**

Cibois, A., E. Pasquet, and T. S. Schulenberg. 1999. Molecular systematics of the Malagasy babblers (Passeriformes: Timaliidae) and warblers (Passeriformes: Sylviidae), based on cytochrome-*b* and 16s rRNA sequences. *Molecular Phylogenetics and Evolution* **13**(3):581–595. doi: 10.1006/mpev.1999.0684

Darriba, D., G. L. Taboada, R. Doallo, and D. Posada. 2012. jModelTest 2: More models, new heuristics and parallel computing. *Nature Methods* **9**(8):772. doi:10.1038/nmeth.2109

Dong, F., S.-H. Li, and X.-j. Yang. 2010a. Molecular systematics and diversification of the Asian scimitar babblers (Timaliidae, Aves) based on mitochondrial and nuclear DNA sequences. *Molecular Phylogenetics and Evolution* **57**(3):1268–1275. doi: 10.1016/j.ympev.2010.09.023

Dong, F., F. Wu, L.-M. Liu, and X.-j. Yang. 2010b. Molecular phylogeny of the barwings (Aves: Timaliidae: Actinodura), a paraphyletic group, and its taxonomic implications. *Zoological Studies* **49**(5):703–709.

Guindon, S., and O. Gascuel. 2003. A simple, fast and accurate method to estimate large phylogenies by maximum-likelihood. *Systematic Biology* **52**(5):696–704. doi: 10.1080/10635150390235520

Hasegawa, M., H. Kishino, and T. Yano. 1985. Dating of the human-ape splitting by a molecular clock of mitochondrial DNA. *Journal of Molecular Evolution* **22**(2):160–174. doi: 10.1007/BF02101694

Librado, P., and J. Rozas. 2009. DnaSP v5: A software for comprehensive analysis of DNA polymorphism data. *Bioinformatics* **25**(11):1451–1452. doi: 10.1093/bioinformatics/btp187

Sorenson, M. D., J. C. Ast, D. E. Dimcheff, T. Yuri, and D. P. Mindell. 1999. Primers for a PCR-based approach to mitochondrial genome sequencing in birds and other vertebrates. *Molecular Phylogenetics and Evolution* **12**(2):105–114. doi: 10.1006/mpev.1998.0602
