# Supplementary material for: Population genetics of the olive‐winged bulbul (Pycnonotus plumosus) in a tropical urban‐fragmented landscape
Source: Ecol Evol. 2015 Dec 10;6(1):78–90. doi: 10.1002/ece3.1832 (PMC4716506; doi:10.1002/ece3.1832)
Supplement: Supplementary file 2 — Figure S1. Haplotype map of Singapore based on ND2 data, showing haplotypes by locality. Table S1. Sample number and collection locality of study samples. Table S2. Matrix of nucleotide diversity, showing π (above diagonal) and p‐divergence (below diagonal) among the five general collecting localities of Singapore (Central Catchment, Central‐west Singapore, South and Southwest Singapore, Northern Singapore, and Northeast offshore islands). Table S3. Analysis of molecular variance (AMOVA) of Singaporean olive‐winged bulbul populations, grouped into five general collecting localities (Central Catchment, Central‐west Singapore, South and Southwest Singapore, Northern Singapore, and Northeast offshore islands). [file ECE3-6-078-s002.docx]

**Supporting Information**


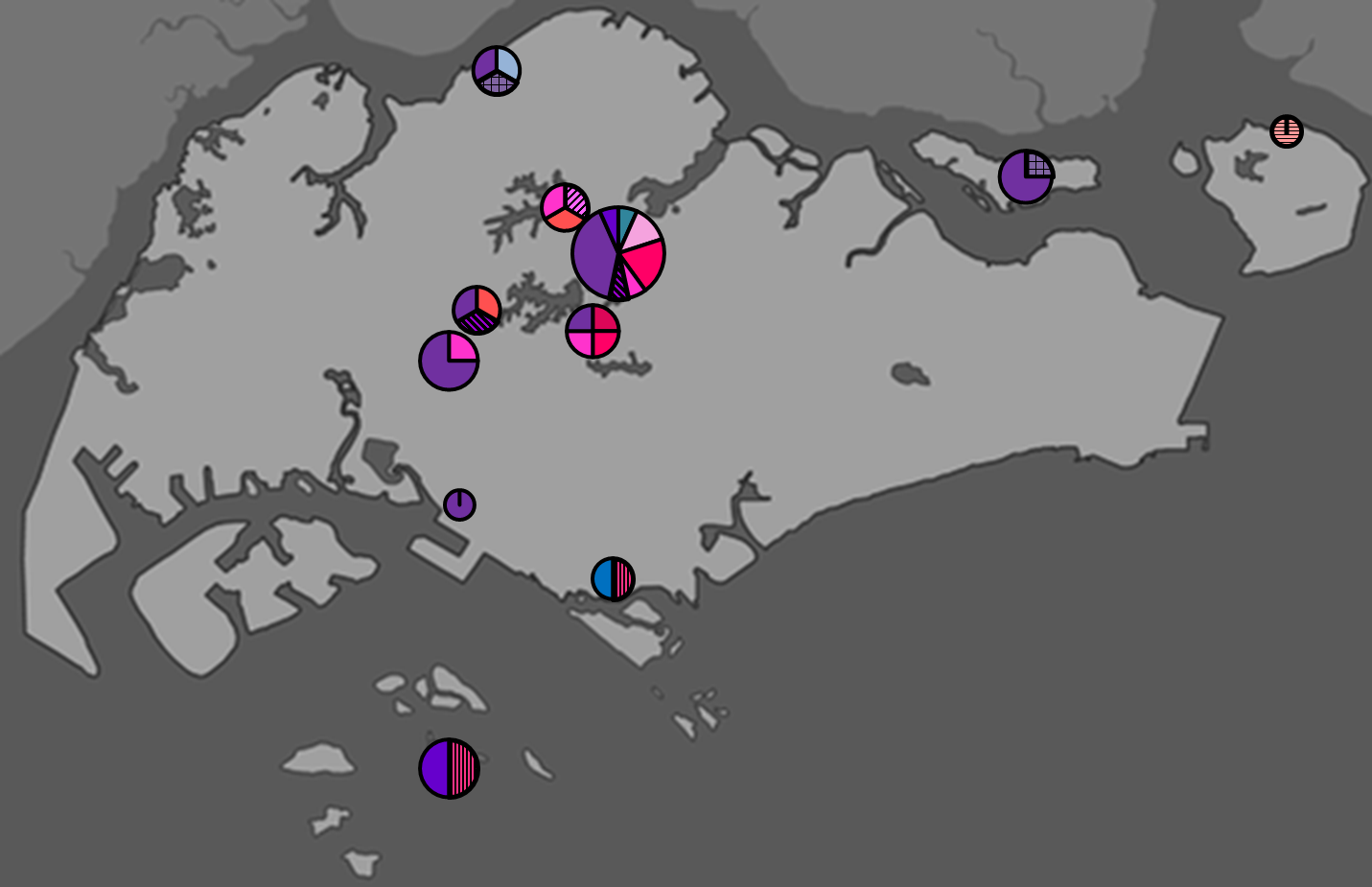


**Figure S1.** Haplotype map of Singapore based on ND2 data, showing haplotypes by locality. Size of circles are proportional to the number of samples from that locality, ranging from n=1 to n=15. Colours that are more similar indicate haplotypes that are more similar in sequence. Seven haplotypes out of 15 are shared across localities, while one particular haplotype (solid purple) is shared among eight of the 11 localities across the island, including offshore localities.

**Table S1.** Sample number and collection locality of study samples. Institutional abbreviations: KUMNH = Kansas University Museum of Natural History; LSUMNS = Louisiana State University Museum of Natural Science; RMBR = Raffles Museum of Biodiversity Research (now renamed the Lee Kong Chian Natural History Museum); SP = Sabah Parks Museum Collection; UNIMAS = University Malaysia Sarawak; UWBM = University of Washington Burke Museum. Detailed sampling locality is given where available. Locality number corresponds to the numerical labels on the sample localities in Figure 1; samples with no locality number were not collected by the authors.

| Genus | Species | Subspecies | Country | State/Province/County/Locality | Locality No. | Sample No. | GenBank Accession No. |
| --- | --- | --- | --- | --- | --- | --- | --- |
| Ingroup taxa | |  |  |  |  |  |  |
| *Pycnonotus* | *Plumosus* | *plumosus* | Singapore | Upper Peirce, Island Service Reservoir |  | CSW7041 | KT321609, KT321532, KT321580 |
|  |  |  | Singapore | MacRitchie Ranger Station | 6 | L1567 | KT321610, KT321533, KT321581 |
|  |  |  | Singapore | Upper Peirce, Hilltop | 3 | M0099 | KT321611, KT321534, KT321582 |
|  |  |  | Singapore | Pulau Ubin | 9 | M0830 | KT321612, KT321535, KT321583 |
|  |  |  | Singapore | Pulau Ubin | 9 | M0831 | KT321613, KT321536, KT321584 |
|  |  |  | Singapore | Pulau Ubin | 9 | M0832 | KT321614, KT321537, KT321585 |
|  |  |  | Singapore | Pulau Ubin | 9 | M0836 | KT321615, KT321538, KT321586 |
|  |  |  | Singapore | MacRitchie Ranger Station | 6 | M0914 | KT321616, KT321539, KT321587 |
|  |  |  | Singapore | Upper Peirce, Hilltop | 3 | M0920 | KT321617, KT321540, KT321588 |
|  |  |  | Singapore | Upper Peirce, Hilltop | 3 | M0921 | KT321618, KT321541 |
|  |  |  | Singapore | Upper Peirce, Hilltop | 3 | M0922 | KT321619, KT321542, KT321589 |
|  |  |  | Singapore | Upper Peirce | 3 | M0923 | KT321620, KT321543, KT321590 |
|  |  |  | Singapore | Upper Peirce | 3 | M0924 | KT321621, KT321544, KT321591 |
|  |  |  | Singapore | Upper Peirce | 3 | M0925 | KT321622, KT321545, KT321592 |
|  |  |  | Singapore | Upper Peirce | 3 | M0926 | KT321623, KT321546 |
|  |  |  | Singapore | Upper Peirce | 3 | M0927 | KT321624, KT321547, KT321593 |
|  |  |  | Singapore | Upper Peirce | 3 | M0928 | KT321625, KT321548 |
|  |  |  | Singapore | Upper Seletar | 2 | M0930 | KT321626, KT321549, KT321594 |
|  |  |  | Singapore | MacRitchie Ranger Station | 6 | M0931 | KT321627, KT321550, KT321595 |
|  |  |  | Singapore | Upper Peirce, Island Service Reservoir | 3 | M0932 | KT321628, KT321551, KT321596 |
|  |  |  | Singapore | Upper Peirce, Island Service Reservoir | 3 | M0933 | KT321629, KT321552, KT321597 |
|  |  |  | Singapore | MacRitchie Ranger Station | 6 | M0934 | KT321630, KT321553, KT321598 |
|  |  |  | Singapore | Upper Peirce, Entrance to Pipeline | 3 | M0935 | KT321631, KT321554 |
|  |  |  | Singapore | Upper Seletar | 2 | M0936 | KT321632, KT321555, KT321599 |
|  |  |  | Singapore | Dairy Farm Nature Park | 4 | M0938 | KT321633, KT321556 |
|  |  |  | Singapore | Dairy Farm Nature Park | 4 | M0940 | KT321634, KT321557, KT321600 |
|  |  |  | Singapore | Dairy Farm Nature Park | 4 | M0941 | KT321635, KT321558 |
|  |  |  | Singapore | Dairy Farm Nature Park | 4 | M0942 | KT321636, KT321559 |
|  |  |  | Singapore | Mount Faber Park | 8 | M1103 | KT321637, KT321560, KT321601 |
|  |  |  | Singapore | Mount Faber Park | 8 | M1104 | KT321638, KT321561, KT321602 |
|  |  |  | Singapore | Bukit Batok Nature Park | 5 | M1105 | KT321639, KT321562, KT321603 |
|  |  |  | Singapore | Bukit Batok Nature Park | 5 | M1106 | KT321640, KT321563, KT321604 |
|  |  |  | Singapore | Bukit Batok Nature Park | 5 | M1107 | KT321641, KT321564 |
|  |  |  | Singapore | Bukit Batok Nature Park | 5 | M1108 | KT321642, KT321565 |
|  |  |  | Singapore | Admiralty Park | 1 | M1109 | KT321643, KT321566, KT321605 |
|  |  |  | Singapore | Admiralty Park | 1 | M1110 | KT321644, KT321567, KT321606 |
|  |  |  | Singapore | Admiralty Park | 1 | M1111 | KT321645, KT321568 |
|  |  |  | Singapore | Clementi Woods Park | 7 | M1112 | KT321646, KT321569, KT321607 |
|  |  |  | Singapore | Upper Peirce, Entrance to Pipeline | 3 | M1113 | KT321647, KT321570 |
|  |  |  | Singapore | Upper Seletar | 2 | M1114 | KT321648, KT321571 |
|  |  |  | Singapore | Pulau Tekong, Unum Hotspring |  | WLK677 | KT321649, KT321572, KT321608 |
|  |  |  | Singapore | Pulau Semakau |  | UWBM 116729 | KT321650 |
|  |  |  | Singapore | Pulau Semakau |  | UWBM 116733 | KT321651, KT321573 |
|  |  |  | Singapore | Pulau Semakau |  | UWBM 116734 | KT321652, KT321574 |
|  |  |  | Singapore | Pulau Semakau |  | UWBM 116736 | KT321653, KT321575 |
|  |  |  | Singapore | Pulau Semakau |  | UWBM 116738 | KT321654, KT321576 |
|  |  |  | Singapore | Pulau Semakau |  | UWBM 116739 | KT321655, KT321577 |
|  |  |  | Singapore | Singapore |  | C704 | HQ011140 |
|  |  |  | Singapore | Singapore |  | L620 | HQ011141 |
|  |  |  | Singapore | Singapore |  | L623 | HQ011142 |
|  |  |  | Singapore | Singapore |  | L970 | HQ011143 |
|  |  |  | Singapore | Singapore |  | L978 | HQ011144 |
|  |  |  | Singapore | Singapore |  | PU10 | HQ011145 |
|  |  |  | Singapore | Singapore |  | PU9 | HQ011146 |
|  |  |  | Singapore | Singapore |  | TL1 | HQ011147 |
|  |  |  | Singapore | Singapore |  | TL15 | HQ011148 |
|  |  |  | Malaysia | Johor |  | LSUMNS 52080 | HQ011139 |
|  |  |  | Malaysia | Sarawak, Bako National Park |  | UWBM 81909 | KT321656, KT321578 |
|  |  |  | Malaysia | Sarawak, Bako National Park |  | UWBM 81934 | HQ011136 |
|  |  |  | Malaysia | Sarawak, Bako National Park |  | UWBM 81936 | KT321657, KT321579 |
|  |  |  | Malaysia | Sarawak |  | LSUMNS B57032 | HQ011131 |
|  |  |  | Malaysia | Sarawak |  | LSUMNS B57047 | HQ011132 |
|  |  |  | Malaysia | Sarawak |  | LSUMNS B57053 | HQ011133 |
|  |  |  | Malaysia | Sarawak |  | LSUMNS B58202 | HQ011134 |
|  |  |  | Malaysia | Sarawak |  | LSUMNS B58218 | HQ011135 |
|  |  |  | Malaysia | Sarawak |  | UNIMAS B1395 | HQ011138 |
|  |  |  | Malaysia | Sabah |  | LSUMNS B23354 | DQ402239 |
|  |  |  | Malaysia | Sabah |  | LSUMNS B46957 | HQ011123 |
|  |  |  | Malaysia | Sabah |  | LSUMNS B46965 | HQ011124 |
|  |  |  | Malaysia | Sabah |  | LSUMNS B46969 | HQ011125 |
|  |  |  | Malaysia | Sabah |  | LSUMNS B47033 | HQ011126 |
|  |  |  | Malaysia | Sabah |  | LSUMNS B47151 | HQ011127 |
|  |  |  | Malaysia | Sabah |  | LSUMNS B47186 | HQ011128 |
|  |  |  | Malaysia | Sabah |  | LSUMNS B51052 | HQ011129 |
|  |  |  | Malaysia | Sabah |  | LSUMNS B51061 | HQ011130 |
| *Pycnonotus* | *Plumosus* | *cinereifrons* | Philippines | Palawan |  | KUNHM 12667 | JN826640 |
|  |  |  | Philippines | Palawan, Puerto Princesa |  | KU 12660 | GU112684 |
|  |  |  | Philippines | Palawan, Mantalingahan Range |  | KU 12806 | GU112685 |
| *Pycnonotus* | *Plumosus* | *hachisukae* | Malaysia | Sabah, Pulau Balambangan |  | SP 22149 | GU112687 |
| *Pycnonotus* | *Plumosus* | *hutzi* | Malaysia | Sabah |  | KU 17707 | GU112686 |
| Outgroup taxa | |  |  |  |  |  |  |
| *Pellorneum* | *malaccense* | *malaccense* | Singapore | Upper Seletar |  | L1807 | KT152765, KT152707, KT152736 |

**Table S2.** Matrix of nucleotide diversity, showing π (above diagonal) and p-divergence (below diagonal) among the five general collecting localities of Singapore (Central Catchment, Central-west Singapore, South and Southwest Singapore, Northern Singapore, and Northeast offshore islands).

|  | Central Catchment | Central-west | South and Southwest | North | Northeast islands |
| --- | --- | --- | --- | --- | --- |
| Central Catchment | – | 0.00261 | 0.00270 | 0.00318 | 0.00271 |
| Central-west | 0.00268 | – | 0.00342 | 0.00486 | 0.00284 |
| South and Southwest | 0.00295 | 0.00325 | – | 0.00486 | 0.00309 |
| North | 0.00538 | 0.00543 | 0.00506 | – | 0.00368 |
| Northeast islands | 0.00290 | 0.00280 | 0.00313 | 0.00436 | – |

**Table S3.** Analysis of molecular variance (AMOVA) of Singaporean olive-winged bulbul populations, grouped into five general collecting localities (Central Catchment, Central-west Singapore, South and Southwest Singapore, Northern Singapore, and Northeast offshore islands).

| Hierarchical structure | Source of variation | Sum of squares (df) | Variance component | *p*-value | Fixation index | Percentage of variation |
| --- | --- | --- | --- | --- | --- | --- |
| 1 | Among  localities | 15.195  (4) | 0.25 | 0.094 |  | 8.48 |
| 2 | Within  localities | 63.771 (24) | 2.66 | N.A. | F_ST_ = 0.08 | 91.52 |
